# Influences of naturally occurring agents in combination with fluoride on gene expression and structural organization of *Streptococcus mutans *in biofilms

**DOI:** 10.1186/1471-2180-9-228

**Published:** 2009-10-28

**Authors:** Jae-Gyu Jeon, Marlise I Klein, Jin Xiao, Stacy Gregoire, Pedro L Rosalen, Hyun Koo

**Affiliations:** 1 Center for Oral Biology, University of Rochester Medical Center, Rochester, New York, USA; 2 Department of Microbiology and Immunology, University of Rochester Medical Center, Rochester, New York, USA; 3 Department of Preventive Dentistry, School of Dentistry and Institute of Oral Bioscience, Chonbuk National University, Jeonju, Republic of Korea; 4 Department of Physiological Sciences, Piracicaba Dental School, University of Campinas (UNICAMP), Sao Paulo, Brazil; 5 Natural Product Research in Oral Biology Group (NatPROB), Piracicaba Dental School, University of Campinas (UNICAMP), Sao Paulo, Brazil

## Abstract

**Background:**

The association of specific bioactive flavonoids and terpenoids with fluoride can modulate the development of cariogenic biofilms by simultaneously affecting the synthesis of exopolysaccharides (EPS) and acid production by *Streptococcus mutans*, which enhanced the cariostatic effectiveness of fluoride *in vivo*. In the present study, we further investigated whether the biological actions of combinations of myricetin (flavonoid), *tt*-farnesol (terpenoid) and fluoride can influence the expression of specific genes of *S. mutans *within biofilms and their structural organization using real-time PCR and confocal fluorescence microscopy.

**Results:**

Twice-daily treatment (one-minute exposure) during biofilm formation affected the gene expression by *S. mutans *both at early (49-h) and later (97-h) stages of biofilm development. Biofilms treated with combination of agents displayed lower mRNA levels for *gtfB *and *gtfD *(associated with exopolysaccharides synthesis) and *aguD *(associated with *S. mutans *acid tolerance) than those treated with vehicle-control (*p *< 0.05). Furthermore, treatment with combination of agents markedly affected the structure-architecture of *S. mutans *biofilms by reducing the biovolume (biomass) and proportions of both EPS and bacterial cells across the biofilm depth, especially in the middle and outer layers (vs. vehicle-control, *p *< 0.05). The biofilms treated with combination of agents were also less acidogenic, and had reduced amounts of extracellular insoluble glucans and intracellular polysaccharides than vehicle-treated biofilms (*p *< 0.05).

**Conclusion:**

The data show that the combination of naturally-occurring agents with fluoride effectively disrupted the expression of specific virulence genes, structural organization and accumulation of *S. mutans *biofilms, which may explain the enhanced cariostatic effect of our chemotherapeutic approach.

## Background

Oral diseases related to dental biofilms, such as dental caries, continue to afflict the majority of the World's population [1]. This ubiquitous disease results from the interaction of specific bacteria with constituents of the diet within a biofilm known as plaque. *Streptococcus mutans *effectively colonizes tooth surfaces, and is a key contributor to the formation of cariogenic biofilms because this bacterium (i) utilizes dietary sucrose to synthesize large amounts of extracellular polysaccharides (EPS), (ii) adheres tenaciously to glucan-coated surfaces, and (iii) is also highly acidogenic and acid-tolerant [2,3].

The majority of biofilm matrices are rich in polysaccharides, and dental biofilms are no exception. Polysaccharides of dental biofilms are mostly glucans synthesized by microbial glycosyltransferases (Gtfs), which are largely insoluble and complex in structure [4,5]. The Gtfs secreted by *S. mutans *(particularly GtfB and GtfC) bind to the tooth surface and to surfaces of bacteria [6-8]. The glucans synthesized by surface-adsorbed Gtfs provide specific binding sites for bacterial colonization on the tooth surface and to each other; thus, contributing to the initial steps of cariogenic biofilm development [3,8]. If the biofilm is allowed to remain on tooth surfaces and is exposed to dietary carbohydrates frequently (especially sucrose), *S. mutans *as a constituent of the biofilm community will continue to synthesize polysaccharides and metabolize the sugars to organic acids. The elevated amounts of EPS, which may involve up-regulation of *gtf *genes in response to pH and carbohydrate availability [9], increase the biofilms bulk and stability, and enhance its virulence [1,3]. In addition, the ability of *S. mutans *to utilize some extra- and intracellular polysaccharides as short-term storage compounds offers an additional ecological benefit, and simultaneously, increases the amount of acid production and the extent of acidification. The persistence of this aciduric environment leads to selection of highly acid tolerant (and acidogenic) flora [1,2,10]; the low pH environment within the biofilm's matrix results in dissolution of enamel, thus initiating the pathogenesis of dental caries. Clearly, EPS (e.g. glucans) and acidification of the matrix by *S. mutans *(and other acidogenic and aciduric organisms) could be primary targets for chemotherapeutic intervention to prevent the formation of cariogenic biofilms.

Strategies of controlling biofilm aimed at disrupting bacterial virulence offer an attractive and alternative approach to the traditional antimicrobial therapy based on use of broad spectrum microbiocides [11]. We have followed a novel combination therapy using specific naturally occurring compounds and fluoride aiming at disrupting EPS-matrix formation and acidogenicity of *S. mutans *within biofilms [12,13]. The strategy is based on their interconnected biological activities; the bioflavonoids (e.g. apigenin or myricetin) are potent inhibitors of glucan synthesis by Gtf enzymes [12,14] whereas the terpenoids(e.g. *tt*-farnesol) and fluoride disrupts the proton permeability of *S. mutans *membrane, affecting its glycolytic activity, production-secretion of Gtfs and acidurance [10,15,16]; fluoride, of course, has additional physicochemical effects [17,18]. The combination of natural agents with 250 ppm fluoride resulted in enhanced cariostatic properties of fluoride *in vivo*, without suppressing the resident oral flora [12,13].

In this study, we further investigated whether the biological actions of the combination of agents can influence the expression of specific genes of *Streptococcus mutans *during biofilm formation, and the spatial distribution of bacterial cells and exopolysaccharides in the biofilm's matrix.

## Methods

### Test compounds

Myricetin was obtained from Extrasynthese Co. (Genay-Sedex, France). *tt*-Farnesol and sodium fluoride were purchased from Sigma-Aldrich Co. (St Louis, MO). For this study, we tested 1.0 mM myricetin and 2.5 mM *tt*-farnesol in combination with sodium fluoride (125 ppm F or 250 ppm F). The concentrations of the natural agents were selected based on data from our previously published and unpublished response to dose studies [13,19,20]. Fluoride at 225-250 ppm is a clinically proven anticaries agent, and is the concentration found in most of the currently commercially available fluoride-based mouth rinses as reviewed in Marinho et al. [17] and Zero [18]. The test agents, including fluoride, were dissolved in 20% ethanol containing 2.5% dimethyl sulphoxide (DMSO) just prior to carrying out the assays.

### Biofilm preparation and treatments

Biofilms of *S. mutans *UA159 were formed on saliva-coated hydroxyapatite (sHA) discs (surface area of 2.93 ± 0.2 cm^2^, Clarkson Chromatography Products Inc., South Williamsport, PA, USA) in batch cultures for 5 days, as detailed elsewhere [21]. The biofilms were grown in ultrafiltered (10 kDa molecular-weight cut-off) buffered tryptone yeast-extract broth containing 1% (w/v) sucrose [21]. The culture medium was replaced daily; the organisms were grown undisturbed for 22 h to allow initial biofilm formation. At this point (22 h old), the biofilms were then treated twice-daily (at 10 a.m. and 4 p.m.) until the end of the experimental period (118-h-old biofilm) with one of the following: (i) 1.0 mM myricetin + 2.5 mM *tt*-farnesol + 125 ppm fluoride (MFar125F); (ii) 1.0 mM myricetin + 2.5 mM *tt*-farnesol + 250 ppm fluoride (MFar250F); (iii) 250 ppm fluoride (250F); (iv) vehicle control (20% ethanol containing 2.5% DMSO in water); fluoride at 125 ppm F was not included because it is devoid of any significant anti-biofilm effects [12,13]. The biofilms were exposed to the treatments for 1 min., dip-rinsed three times in sterile saline solution (to remove excess of agents or vehicle-control) and transferred to culture medium. The treatments and rinsing procedures were repeated 6 h later. The pH of culture medium surrounding the biofilms was also determined during the experimental period (until 118 hour biofilms, at 8 a.m., 12 a.m., 4 p.m., 6 p.m.). Our previous studies have shown that the vehicle control (1 min exposure, twice daily) allowed the continued formation of biofilm, and did not affect the biochemical composition and cell viability when compared to biofilms treated with saline solution [20,21]. Each biofilm was exposed to the respective treatment a total of 8 times. Biofilm assays were performed in duplicate in at least six different experiments.

### RNA extraction and real-time RT-PCR

At selected time points (49- and 97-h-old biofilms), RNA was extracted and purified using standard protocols optimized for biofilms [22]; RNA integrity number (RIN) for our samples was ≥ 9.0 as determined by lab on-chip capillary electrophoresis [22]. The reverse transcriptase PCR, real-time qPCR amplification conditions, and the gene-specific primers (for *gtfB, gtfC *and *gtfD*) were similar to those described previously [14]. Specific genes related to acid tolerance mechanisms, *aguD *(part of the agmatine deiminase system operon) and *atpD *(part of the F-ATPase operon) were also tested. The *aguD *(5- ATCCCGTGAGTGATAGTATTTG -3 and 5-CAAGCCACCAACAAGTAAGG-3) and *atpD *(5-CGTGCTCTCTCGCCTGAAATAG-3 and 5-ACTCACGATAACGCTGCAAGAC-3) specific primers were designed using Beacon Designer 2.0 software (Premier Biosoft International, Palo Alto, CA, USA). Briefly, cDNAs were synthesized using BioRad iScript cDNA synthesis kit (Bio-Rad Laboratories, Inc., CA). To check for DNA contamination, purified total RNA without reverse transcriptase served as a negative control. The resulting cDNA and negative controls were amplified by a MyiQ real-time PCR detection system with iQ SYBR Green supermix (Bio-Rad Laboratories, Inc., CA, USA) and specific primers. A standard curve was plotted for each primer set as detailed elsewhere [14]. The standard curves were used to transform the critical threshold cycle (Ct) values to the relative number of cDNA molecules. Relative expression was calculated by normalizing each gene of interest of the treated biofilms to the 16SrRNA gene, which served as the reference gene [14]. These values were then compared to those from biofilms treated with vehicle-control to determine the change in gene expression [14]. The number of copies of 16SrRNA in the biofilms treated with test agents and vehicle control was not significantly different from each other (*P *> 0.05).

### Laser scanning confocal fluorescence microscopy imaging of biofilms

At the end of the experimental period (118-h-old biofilms), the structural organization of the biofilms was examined by simultaneous *in situ *labeling of extracellular polysaccharides (EPS) and bacterial cells as described by Klein *et al*. [23]. Briefly, 2.5 μM of Alexa Fluor^® ^647-labeled dextran conjugate (10,000 MW; absorbance/fluorescence emission maxima 647/668 nm; Molecular Probes Inc., Eugene, OR) were added to the culture medium during the formation and development of *S. mutans *biofilms. The fluorescence-labeled dextran serves as a primer for Gtfs and can be simultaneously incorporated during the extracellular polysaccharide matrix synthesis over the course of the biofilm development, but does not stain the bacterial cells at concentrations used in this study [23]. The bacterial cells in biofilms were labeled by means of 2.5 μM of SYTO^® ^9 green-fluorescent nucleic acid stain (480/500 nm; Molecular Probes Inc., Eugene, OR) using standard procedures [24,25]. Laser scanning confocal fluorescence imaging of the biofilms was performed using a Leica TCS SP1 microscope (Leica Lasertechnik, GmbH, and Heidelberg, Germany) equipped with argon-ion and helium neon lasers tuned to 488 and 633 nm, respectively. Triple dichroic (488/543/633) and emission filters (Chroma Technology Corp., Rockingham, VT) were selected for detection of Alexa Fluor^® ^647 and SYTO^® ^9. Confocal images were acquired using a 40×, 0.8 numerical aperture water-immersion objective lens, which provided an optical section thickness of approximately 1 μm. Each biofilm was scanned at 5 randomly selected positions, and z series were generated by optical sectioning at each of these positions. Images were constructed from a 512 × 512 array of pixels spanning a 250 μm field of view (FOV).

### Image analysis

Three independent biofilm experiments were performed and 5 image stacks (512 × 512 pixel tagged image file format) per experiment were collected [23]. The biofilm structure was quantified from the confocal stacks by the image-processing software COMSTAT [26]. For this study, biovolume and area occupied by bacteria and polysaccharides in each layer were utilized to determine the differences among biofilms treated with the various test agents and control. The biovolume is defined as the volume of the biomass (μm^3^) divided by substratum (HA surface) area (μm^2^). The area occupied by bacteria and polysaccharides in each layer indicates the fraction (in percentage) of the area occupied by either components in each image of a stack, and provides the vertical distribution of each of the biofilm components (from deeper to outer regions of the biofilm. The three-dimensional architecture of the biofilms was visualized using Amira™ 4.1.1 (Mercury Computer Systems Inc., Chelmsford, MS, USA).

### Biochemical analyses

The biochemical composition of the biofilms (118-h) were also determined [21,27]. The biofilms were removed and subjected to sonication using three 30-s pulses at an output of 7 W (Branson Sonifier 150; Branson Ultrasonics, Danbury, CT) [27]. The homogenized suspension was analyzed for dry-weight, total protein (by acid digestion followed by ninhydrin assay; [28]) and polysaccharide composition. The extracellular water soluble and insoluble glucans, and intracellular iodophilic polysaccharides were extracted and quantified by colorimetric assays as detailed by Koo *et al*. [21].

Furthermore, F-ATPase activity of the treated biofilms was measured according to Belli *et al*. [29]. Briefly, the homogenized suspension was permeabilized by subjecting the biofilm cells to 10% toluene (v/v) followed by two cycles of freezing and thawing. F-ATPase activity was measured in terms of the release of phosphate in the following reaction mixture: 75.0 mmol of Tris-maleate buffer (pH 7.0) containing 5.0 mM ATP, 10.0 mmol MgCl_2 _and permeabilized biofilm cells. The released phosphate (over the 10-min reaction time) was determined by the method of Bencini *et al*. [30].

### Statistical analyses

The data were analyzed by analysis of variance (ANOVA) in the Tukey-Kramer Honest Standard Deviation (HSD) test for all pairs. Statistical software JMP version 3.1 (SAS Institute, Cary, NC, USA) was used to perform the analyses. The level of significance was set at 5%.

## Results

### Gene expression profile of *S. mutans *biofilms after treatments

**Figure 1:**
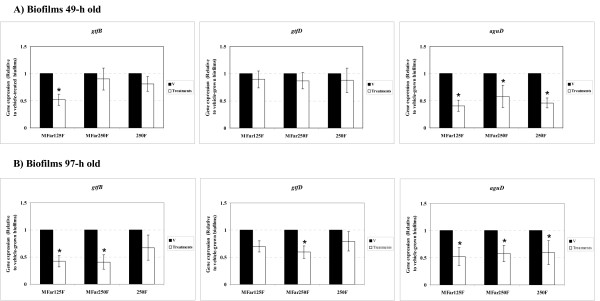
**Real-time PCR analysis of *gtfB*, *gtfD *and *aguD *gene expression by *S. mutans *treated with the test agents**. **A) Biofilms 49-h old; B) 97-h old**. The mRNA level of each gene in each sample was normalized to that of 16S rRNA. These values were then compared to those from vehicle-treated biofilms (V) (corresponding to an arbitrary value of 1) to determine the change (n-fold) in gene expression. Data are expressed as means ± standard deviations of triplicates from at least three separate experiments; values marked with an asterisk are significantly different from that for the vehicle-treated biofilms (*p *< 0.05, ANOVA, comparison for all pairs using Tukey test).

The expression profile of *gtfB, gtfC *and *gtfD *(genes associated with EPS-matrix synthesis), and *aguD *and *atpD *(associated with acid-tolerance) in *S. mutans *biofilms treated with the test agents was determined at two distinct time points (49-h and 97-h) (Figure 1). These two time points represent the early and late stages of biofilm development using our model [[23]; Xiao and Koo, unpublished data].

At 49-h of biofilm development (Figure 1-A), the expression of *gtfB *in MFar125F-treated biofilms was significantly decreased when compared to vehicle-treated biofilms (*p *< 0.05); the expression of other *gtf *genes was unaffected (*p *> 0.05). At 97-h (Figure 1-B), the combination of agents repressed the expression of *gtfB *(by MFar125F and MFar250F) and *gtfD *(MFar250F), but not *gtfC *(data not shown). The expression of *aguD *was significantly reduced by all treatments compared to vehicle-control group at both time points (*p *< 0.05); the expression of *atpD *was unaffected (*p *> 0.05). The transcriptional responses of *S. mutans *to the agents during the course of biofilm development may affect the structural organization and biochemical composition of the biofilms after treatments, which were examined as follows.

### Influences of treatments on structural organization and composition of *S. mutans *biofilms *in vitro*

#### LSCFM imaging and COMSTAT analysis of biofilm constituents

In this study, we determined the biovolume (biomass) and the spatial distribution of extracellular polysaccharides (EPS) and bacterial cells in the biofilms. Our confocal microscopy imaging approach allows for simultaneous quantification and visualization of bacterial cells and EPS, which provide a more precise examination of the biofilm architecture than labeling bacteria alone.

The biovolumes of EPS and bacterial cells of the biofilms treated with combinations of myricetin and *tt*-farnesol with 125 or 250 ppm fluoride (MFar125F and MFar250F) were significantly lower than those of biofilms treated with fluoride alone (250F) or vehicle-control (*p *< 0.05; Table 1).

**Table 1:** Biovolume of *S. mutans *UA159 biofilms after treatments by COMSTAT analysis.

Treatments*	MFar125F		MFar250F		250F		Vehicle control	
**Biofilm components**	**Bacteria**	**EPS**	**Bacteria**	**EPS**	**Bacteria**	**EPS**	**Bacteria**	**EPS**

**Biovolume**	6.3 ± 1.6 A	8.8 ± 2.0 ^δ^	5.4 ± 1.0 A	9.3 ± 0.9 ^δ^	12.3 ± 3.5 B	13.2 ± 0.9 ^ε^	12.0 ± 6.7 B	15.0 ± 5.7 ^ε^

Values (SD, n = 15) in the same line for bacteria followed by the same letters are not significantly different from each other (*p *> 0.05, ANOVA, comparison for all pairs using Tukey test).

Values (SD, n = 15) in the same line for EPS followed by the same symbols are not significantly different from each other (*p *> 0.05, ANOVA, comparison for all pairs using Tukey test).

MFar125F - myricetin, *tt*-farnesol and 125 ppm F; MFar250F - myricetin, *tt*-farnesol and 250 ppm F; 250F - 250 ppm F; Vehicle control - 20% ethanol containing 2.5% DMSO (v/v).

**Figure 2:**
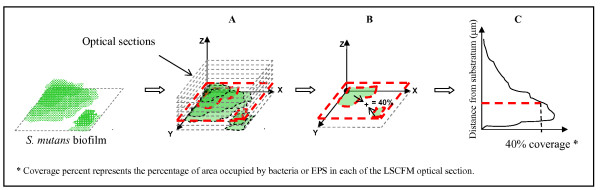
**Schematic diagram of determination of vertical distribution of bacteria or EPS from LSCFM imaging data by COMSTAT**. (A) highlight of an optical section of specific area of the biofilm; (B) COMSTAT calculate the percentage of area occupied by bacteria or EPS on each optical section individually (as highlighted); (C) Then, the data of each optical section is plotted in a graph.

**Figure 3:**
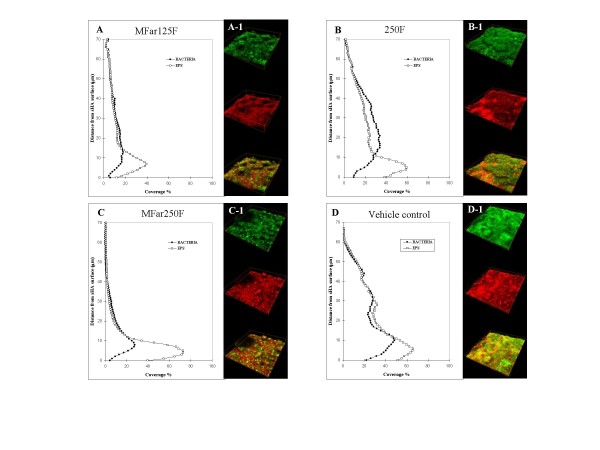
**(A-D) Profile of the distribution of bacteria and EPS in each of the biofilms after treatments (n = 15); (A1-D1) Representative 3-D image of the structural organization of the treated-biofilms**. Bacteria (green) and EPS (red).

The vertical distribution of EPS and bacteria from disc surface to fluid phase was also calculated from the three-dimensional confocal imaging data sets as explained in the schematic diagram in Figure 2. Figure 3(A-D) shows the distribution of both EPS and bacterial cells in the biofilms after treatments. The biofilms treated with the combination of agents exhibited less EPS and bacteria across the biofilm depth, especially in the middle (20 to 40 μm from substratum) and outer layers (above 40 μm), than those treated with 250F or vehicle-control. Furthermore, a representative three-dimensional rendering of bacteria (in green) and EPS (in red) in each of the treated biofilms are shown in Figure 3(A1-D1). Treatments with the combination of agents resulted in biofilms displaying markedly distinctive structure-architecture, which were less compact and less dense (Figure 3A1, and 3C1) compared to those treated with vehicle-control or 250F (Figure 3B1 and 3D1).

#### Biofilm composition analysis of the treated biofilms

**Figure 4:**
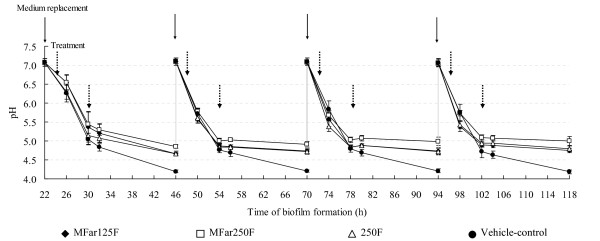
**Influence of treatments on the pH values in the culture medium during *S. mutans *biofilm formation**. The medium was replaced daily with fresh medium. The pH values (n = 9) were determined at 0 h and after 4, 8, 10 and 24 h of incubation each day. Values from vehicle control are significantly different from MFar250 at 10 h and 24 h of incubation, and from all treatments at 24 h of incubation during the entire experimental period (*P *< 0.05, ANOVA, comparison for all pairs using Tukey's test).

Topical applications of combinations of agents resulted in biofilms with significantly less biomass (dry-weight), and total amounts of extracellular insoluble glucans and intracellular (IPS) polysaccharides compared to those treated with vehicle-control (Table 2; *p *< 0.05); MFar250F also diminished the amounts of soluble glucans (vs. vehicle-control; *p *< 0.05). Fluoride treatments also reduced the dry-weight, and markedly disrupted IPS accumulation in the biofilms (vs. vehicle-control; *p *< 0.05), but did not reduce significantly the amounts of exopolysaccharides. Interestingly, biofilms treated with combinations of agents or 250F showed higher levels of F-ATPase activity compared to vehicle-control treated biofilms (*p *< 0.05; Table 2). Furthermore, treatments with combination of agents or 250F also reduced acidogenicity of the biofilms (Figure 4).

**Table 2:** Biomass (dry-weight) and polysaccharides composition in *S. mutans *UA159 biofilms after treatments.

Treatments*	Dry-weight (mg)	Polysaccharides			F-ATPase activity**
			
		Insoluble (μg)	Soluble (μg)	IPS (μg)	
**MFar125F**	3.22 ± 0.68 A	0.92 ± 0.33 A	0.24 ± 0.05 A, B	0.17 ± 0.02 A	0.94 ± 0.30 A
**MFar250F**	3.37 ± 0.55 A	0.98 ± 0.20 A, B	0.22 ± 0.06 A	0.15 ± 0.03 A	1.04 ± 0.27 A
**250F**	4.50 ± 0.48 B	1.33 ± 0.23 B, C	0.24 ± 0.08 A, B	0.18 ± 0.03 A	0.94 ± 0.19 A
**Vehicle control**	5.90 ± 0.80 C	1.70 ± 0.25 C	0.30 ± 0.04 B	0.47 ± 0.06 B	0.52 ± 0.08 B

Values (SD, n = 12) in the same column followed by the same letters are not significantly different from each other (*p *> 0.05, ANOVA, comparison for all pairs using Tukey test).

IPS -- Iodophilic intracellular polysaccharides

* MFar125F - myricetin, *tt*-farnesol and 125 ppm F; MFar250F - myricetin, *tt*-farnesol and 250 ppm F; 250F - 250 ppm F; Vehicle control - 20% ethanol containing 2.5% DMSO (v/v).

** Expressed as μg of phosphate released/mg of protein

## Discussion

Development of novel chemotherapeutic approaches, other than microbiocides, that disrupt the establishment, structure and virulence of dental biofilms could be a promising route to prevent or reduce the pathogenesis of oral infectious diseases such as dental caries. Currently, fluoride in various preparations is the mainstay for caries prevention [31]. Fluoride exerts its major effects by reducing enamel-dentine demineralization and enhancing remineralization of early caries lesions [18]. However, fluoride, at levels found in plaque, also displays biological effects on critical virulence factors of cariogenic streptococci, particularly (albeit not exclusively) on *S. mutans *[10]. Nevertheless, as currently used, fluoride offers incomplete protection against dental caries (18). Thus, any agent that enhances its protective effects clearly has clinical potential. Recently, we have identified specific flavonoids (myricetin) and terpenoids (*tt*-farnesol) that exhibit bioactivity against *S. mutans; *these compounds are ubiquitously found in fruits (cranberries and red wine grapes) and propolis (a beehive product) [12,13,19,20]. The concentrations of 1.0 mM myricetin and 2.5 mM *tt*-farnesol displayed the most potent inhibitory effects on glucans synthesis and acid production by *S. mutans *cells as determined from our published and unpublished response to dose studies [13,19,20]. Furthermore, the combination of the naturally occurring agents with 250 ppm fluoride was the most effective in reducing *S. mutans *biofilm formation and EPS synthesis *in vitro*, and also enhanced cariostatic properties of fluoride *in vivo *[12,13].

Analysis of our data shows that the natural agents acting in concert with fluoride (at 125 or 250 ppm) modulated the expression of specific virulence genes by *S. mutans*, and also disrupted the accumulation and structural organization of extracellular polysaccharides (EPS) and bacterial cells in the matrix, which affected the biochemical and physiological properties of the biofilms *in vitro*. Although our mono-species biofilm model does not mimic exactly the complex microbial community found in coronal dental plaque, it does however place emphasis on a critical virulence characteristic of the biofilm, i.e. the polysaccharide matrix. Furthermore, biofilms using a single organism is advantageous in examining the mechanisms of actions of therapeutic agents on *S. mutans *physiology and genetics, especially on the glucan-mediated processes involved in the formation of the polysaccharide matrix in biofilm.

Our *in vitro *data suggest at least two major mechanisms of actions by which the combination therapy affects *S. mutans *virulence: (1) inhibition of insoluble exopolysaccharides synthesis, particular by GtfB, and (2) reduction of intracellular polysaccharide accumulation and aciduricity associated with cytoplasmic acidification and starvation stress. The combination of agents, especially MFar125F, markedly reduced the *gtfB *mRNA levels in *S. mutans *biofilms both at early and later stages of biofilm development. The reduction of *gtfB *expression in addition to inhibitory effects on GtfB activity (by myricetin; [19]) and enzyme production-secretion (by fluoride and *tt*-farnesol; [16,21]) appear to be one of the main pathways in altering the accumulation and structure of biofilms. We have shown previously that brief exposure (one-minute) of biofilms to 2.5 mM *tt*-farnesol and 1 mM myricetin had negligible effects on the vitality of *S. mutans *in biofilms (compared to either vehicle treated or untreated biofilms) [12,13,21]. In this study, the combinations of agents with fluoride were devoid of any significant bactericidal activity against biofilms under our experimental conditions.

GtfB secreted by *S. mutans *not only binds to the apatitic surface, but also on the bacterial surface in an active form [8], which are advantageous to the organisms for the persistent colonization of tooth surfaces [3]. The disruption of insoluble glucans synthesis *in situ *would contribute to (i) the overall decrease of the exopolysaccharide content and bacterial biomass, and (ii) may explain lower EPS biovolume within the biofilms' matrix after treatments with the combination therapies. Biofilms containing lower amounts of insoluble glucans across the depth of the biofilms could influence the pathogenesis by disrupting physical integrity and stability [32], affecting the diffusion properties [33], and reducing the binding sites for mutans streptococci and lactobacilli [3,8]. The altered tridimensional structure-architecture containing less insoluble-glucans may also be more susceptible to inimical influences of antimicrobials and other environmental assaults [34].

Furthermore, *gtfB *gene is a recognized virulence factor associated with the pathogenesis of dental caries in rodents [35]; mutant strains of *S. mutans *defective in *gtfB *are far less cariogenic than parent strains *in vivo*, particularly on smooth-surface caries [35]. Higher content of insoluble glucans in the matrix is associated with increased cariogenicity of biofilms in humans [36]. A recent study showed that GtfB levels in saliva correlated with presence of clinical caries in humans [37]. Thus, the combination therapy would result in a less virulent (cariogenic) biofilm. In addition, the expression of *gtfD *was also repressed by MFar250F in the biofilms at later stages of development (97-h-old); the soluble glucans produced by GtfD can serve as primer for insoluble glucan synthesis, and can be metabolized into acids by *S. mutans *[3,35], which are additional routes for expression of virulence by this bacterium. Further studies using functional genomics approaches shall elucidate the exact mechanisms by which the combination of agents affects the transcription of these critical genes.

Concomitantly, marked reductions in IPS accumulation and enhanced F-ATPase activity along with repression of *aguD *gene expression may indicate disruption of ΔpH across the cell membrane and energy starvation [21] in the biofilms-cells treated with the test agents. Aciduric bacteria such as the mutans streptococci can carry out glycolysis at low pH values within the biofilm's matrix even though glycolytic enzymes are not acid tolerant, because the bacteria maintain ΔpH across the cell membrane with the interior more alkaline than the exterior. During glycolysis, protons are moved out of the cell through the proton-translocating, membrane F-ATPase.

Fluoride short circuits this flow through the diffusion of HF into cell, which acidifies the cytoplasm, inhibits intracellular enzymes and greatly reduces the ATP-pools in biofilm-cells [10,16]. By increasing re-entry of protons across the cell membrane, it increases the demand on ATP that is used by F-ATPase to pump-out protons for acid-base regulation compromising the energy status of the cell [10,16]. *tt*-Farnesol and myricetin also contributes to these effects by increasing proton permeability, and inhibiting glycolytic activity [19,21] enhancing the starvation and acid sensitization of the biofilms. Moreover, the repression of *aguD *expression, an important component of the agmatine deiminase system (AgDS), by the agents may augment the starvation stress. AgDS system converts agmatine to putrescine, ammonia and CO_2_; the production of ammonia from agmatine contributes in increasing the cytoplasmic pH and generating ATP that can be used for growth or to extrude protons [38].

Thus, the net result would be cytoplasmic acidification and diminished ATP pools, and thereby disruption of IPS synthesis and acid-tolerance by *S. mutans *within biofilms. The IPS, a glycogen-like storage polymer, provide *S. mutans *with an endogenous source of carbohydrate which can be metabolized when exogenous fermentable substrate have been depleted in the oral cavity; as a result, IPS can promote the formation of dental caries in animals and in humans by prolonging the exposure of tooth surfaces to organic acids and a concomitant lower fasting pH in the matrix of the plaque [39-41]. Clearly, the combined effects of the agents affected acidurance (and acid production) of the biofilms as indicated by higher final pH values of the surrounding medium when compared to control group, particularly MFarF250 treatments. It is noteworthy that agents that act to restrict ATP supply to anabolism and to maintain ΔpH would also affect protein synthesis-secretion and gene expression.

The overall biological effects of the combination therapy, particularly on EPS and IPS synthesis, could affect dramatically the ability of *S. mutans *to colonize on the tooth surfaces and become dominant and express virulence in plaque without necessarily killing the target organism or disrupting the resident flora. This observation is congruent with our previous findings showing effective cariostatic activity of combination of agents without influencing the microbial composition of the animals' plaque in a rat model of dental caries [12,13], which is *de facto *an *in vivo *multispecies system.

It is noteworthy that the combination of natural agents with lower concentration of fluoride (125 ppmF) was highly effective in disrupting biofilms and expression of *gtfB*, which is an indication that may affect caries development *in vivo*. Interestingly, MFar125F was more effective in reducing *gtfB *expression than MFar250F, which could also explain the lower amounts of EPS in the inner layers of the biofilms treated with MFar125F (vs. MFar250F). Additional studies using microarrays shall determine if other genes associated with *gtfB *regulation are differentially affected between MFar125F or MFar250F treatments, and thereby assist us in elucidating the mechanistic basis for the phenomenon observed in this study. At the same time, we are also investigating whether the combination of agents may result in preparations with lower concentrations of fluoride without reducing the cariostatic effectiveness.

## Conclusion

The combined actions of the natural agents and fluoride on (i) production of specific bacterial-derived GtfB glucans and acidogenicity at transcriptional and physiological levels, in addition to (ii) the physico-chemical effects of fluoride may explain the superior cariostatic effect *in vivo *of the combination therapy compared to 250 ppm fluoride or CHX [12,13], which are proven anti-caries/anti-plaque chemical modalities. Further studies using multispecies biofilm models shall elucidate the biological effects of the combination therapy on complex ecological interactions and their influences in the EPS-matrix development, which will advance our understanding of the exact mechanisms of action of these agents.

## Abbreviations

EPS: extracellular polysaccharides; Gtfs: glycosyltransferases; DMSO: dimethyl sulphoxide; HA: hydroxyapatite; sHA: saliva-coated hydroxyapatite; *aguD*: amino acid permease/putrecine antiporter (part of the Agmatine Deiminase System operon); *atpD*: ATPase beta subunit (part of the F-ATPase operon); MFar125F: 1.0 mM myricetin + 2.5 mM *tt*-farnesol + 125 ppm fluoride; MFar250F: 1.0 mM myricetin + 2.5 mM *tt*-farnesol + 250 ppm fluoride; 250F: 250 ppm fluoride; LSCFM: Laser scanning confocal fluorescence microscopy; FOV: field of view.

## Authors' contributions

JGJ planed and carried out the biofilm experiments and the biochemical assays, and also assisted with the data analysis and drafted the manuscript. MIK carried out all the molecular genetic studies and collected, organized and analyzed the real-time PCR data. JX conducted all the LSCFM studies, including image acquisition, data collection and analysis. PLR organized the data, helped to draft the manuscript and revised it for important intellectual content. HK conceived the study, participated in its design and coordination, and was involved in drafting the manuscript and revising it critically for intellectual content. All authors read and approved the manuscript.
